# Genome-oriented treatment strategies for autoimmune diseases

**DOI:** 10.1186/s41232-021-00179-2

**Published:** 2021-09-23

**Authors:** Yoshiya Tanaka

**Affiliations:** The First Department of Internal Medicine, School of Medicine, University of Occupational and Environmental Health, 1-1 Iseigaoka, Kitakyushu, 807-8555 Japan

Autoimmune diseases are defined as conditions in which various organs are damaged by activated autoreactive T cells involved in the failure of immune tolerance or autoantibodies produced by B cells. Organ-specific autoimmune diseases, including ulcerative colitis, Hashimoto’s thyroiditis, multiple sclerosis, and systemic autoimmune diseases that affect and damage multiple organs, such as the skin, joints, heart, kidneys, serosa, nerves, and blood vessels, are also referred to as connective tissue diseases. Rheumatoid arthritis is a typical systemic autoimmune disease affecting most patients, but no specific autoantigens have been identified [1–3]. Various disease-susceptibility genes have been identified, including *HLA-DRB1*, *PTPN22*, *CTLA4*, *STAT4*, *TNFAIP3*, *CCL21*, and *PADI4*. It has been established that when these genes are combined with environmental factors and epigenetically modified through citrullination of extracellular matrix molecules such as filaggrin and fibrinogen, immune tolerance to antigens fails, thereby inducing autoimmunity. Activated lymphocytes produce inflammatory cytokines, such as tumor necrosis factor (TNF) and interleukin (IL)-6, which cause inflammation in multiple organs, including joints, irreversible structural damage to joints due to prolonged inflammation, and multi-organ disorders such as the lung. Joint injury progresses soon after onset, and deformed joints cause irreversible physical dysfunction. Therefore, prompt and appropriate diagnosis and treatment are necessary.

In the 20th century, corticosteroids and non-steroidal anti-inflammatory drugs were used to relieve pain and swelling in multiple joints. However, these drugs cannot control the progression of joint destruction. The consequent issue for treatment was to control endocrine and metabolic adverse drug reactions to the long-term use of corticosteroids and infection associated with susceptibility to infection due to immunosuppression. In the 21st century, the advent of drugs targeting important molecules involved in the pathogenesis of rheumatoid arthritis has brought a paradigm shift in treatment. In addition to the conventional synthetic antirheumatic drugs such as methotrexate, several new drugs have been developed. These include biological antirheumatic drugs purified from biological drugs to target TNF, IL-6, T cell co-stimulation molecule CD28, and others, and targeted synthetic antirheumatic drugs such as Janus kinase inhibitors that are involved in signal transduction of cytokines and others. Clinical remission has become a realistic therapeutic goal for most patients. The maintenance of remission has allowed the prevention of structural joint damage and control of the progression of physical dysfunction over a long period [1–3]. These drugs, approved for treating rheumatoid arthritis, are being used to treat many autoimmune diseases.

Systemic lupus erythematosus (SLE) is a typical systemic autoimmune disease. It commonly occurs in women of childbearing age, affects systemic organs such as the skin, joints, kidneys, serosa, nerves, heart, and blood vessels, and manifests various clinical symptoms. In the past, nonspecific treatment with corticosteroids, immunosuppressants, and other drugs, has been applied to suppress immune abnormalities and prevent the progression of organ dysfunction. Although these drugs have improved the prognosis of this disease, their long-term use causes various adverse reactions. In particular, severe and opportunistic infections associated with susceptibility to infection due to immunosuppression are the leading causes of death from this disease. The development of molecular target drugs has also been attempted for this disease. Its main pathological characteristics are the activation of B cells and the production of autoantibodies due to autoimmune abnormalities [4–6]. B cells are stimulated by T follicular helper cells and autoreactive T cells and then undergo class switching to differentiate into antibody- and autoantibody-producing cells. In addition, B cells express major histocompatibility complex molecules that function as antigen-presenting cells to T cells and other immune cells. Furthermore, B cells are stimulated by the immune system to produce various cytokines. In other words, B cells play a central role as responders and stimulators of the immune system and autoimmune pathology. Thus, B cell-targeting therapy is expected to be the mainstay of treatment for SLE. However, treatment with B cell-targeting biological drugs, such as rituximab and epratuzumab, has not been successful to date. In contrast, many disease-susceptibility genes for SLE that have been identified by genome-wide association analysis exist in B cells [6, 7]. This suggests that this disease is associated with abnormalities in the adaptive immune system.

However, IRF5, IRAK1, TLR7, and other central genes of the innate immune system that are served mainly by dendritic cells have also been identified as disease-susceptibility genes for SLE. In patients with SLE, dendritic cells highly express Toll-like receptors, which bind to stimuli, such as DNA and RNA released during apoptosis and necrosis, to activate the innate immune system. Different cytokines produced by dendritic cells, such as type I interferon (IFN), soluble B cell-activating factor (BAFF), IL-12/IL-23, and chemokines, induce the differentiation and activation of B and T cells in the adaptive immune system. Several successful outcomes have been achieved with biological drugs targeting cytokines, such as soluble BAFF and type I IFN, which are produced by dendritic cells and bridge the innate and adaptive immune systems. This finding has important implications [5–8]. This can also be regarded as a good example of genome-wide association analysis that provides a new understanding of the pathology and new treatment concepts. In SLE, drugs targeting molecules involved in pathogenesis are insufficient, suggesting that the disease cannot be treated without developing a therapeutic approach based on genomic data. Fujio et al. suggested in “Review: transcriptome and trans-omics analysis of systemic lupus erythematosus” that comprehensive analysis of multi-level omics data on not only individual molecules but also the genome, epigenome, transcriptome, proteome, metabolome, etc., in other words, multi-omics or trans-omics analyses lead to the elucidation of new pathological conditions and the discovery of new therapeutic targets for SLE [9].

Such approaches should also be applicable to other autoimmune diseases. In “Roles of cytotoxic lymphocytes and MIC/LILR families in pathophysiology of Takayasu arteritis,” Yoshifuji and Terao reviewed the potential of new therapeutic targets identified for Takayasu arteritis by genome analysis [10]. In “Updates on genetics in systemic sclerosis,” Ota and Kuwana explain the new understanding based on genome analysis in the pathology of systemic sclerosis. Moreover, despite the dramatic advances in the treatment of rheumatoid arthritis, the differentiation of the use of many molecular target drugs is unknown [11]. In “Genomics-driven drug discovery based on disease susceptibility genes,” Sonehara and Okada mention the differential use of molecular target drugs for rheumatoid arthritis based on genome analysis. The introduction of precision medicine using molecular target therapy should lead to efficient treatment, reduce health care costs, and alleviate adverse events. This feature issue presents the latest findings on autoimmune diseases from world-leading researchers in genomic medical studies [12]. Using genomics, multi-omics, and trans-omics data to their fullest extent will bring a new paradigm shift in treating autoimmune diseases, which have been regarded as intractable. This new fledgling trend may develop extensively with various studies in the near future.

## Data Availability

Not applicable.

## Abbreviations

BAFF: B cell-activating factor
IFN: Interferon
IL: Interleukin
SLE: Systemic lupus erythematosus
TNF: Tumor necrosis factor
